# The Heart’s Function as a Pump Assessed via Impedance Cardiography and the Autonomic System Balance in Patients with Early-Stage Acromegaly

**DOI:** 10.3390/jcm13020395

**Published:** 2024-01-11

**Authors:** Agnieszka Jurek, Paweł Krzesiński, Robert Wierzbowski, Beata Uziębło-Życzkowska, Przemysław Witek, Grzegorz Zieliński, Anna Kazimierczak, Małgorzata Banak, Grzegorz Gielerak

**Affiliations:** 1 Department of Cardiology and Internal Medicine, Military Institute of Medicine–National Research Institute, 04-141 Warsaw, Poland; pkrzesinski@wim.mil.pl (P.K.);; 2 Department of Internal Medicine, Endocrinology, and Diabetology, Medical University of Warsaw, 02-091 Warsaw, Poland; 3 Department of Neurosurgery, Military Institute of Medicine–National Research Institute, 04-141 Warsaw, Poland; gzielinski@wim.mil.pl

**Keywords:** acromegaly, cardiovascular complications, heart rate variability, hemodynamic disorders, impedance cardiography

## Abstract

Background: Acromegaly is a rare, chronic disease that involves structural and functional abnormalities of the cardiovascular system. Acromegaly likely affects interactions between the cardiovascular system and the autonomic nervous system (ANS). Therefore, assessing the relationship between sympathetic–parasympathetic balance by analyzing heart rate variability (HRV) and the hemodynamic profile via impedance cardiography (ICG) may be useful in learning the exact nature of interactions between the ANS and the cardiovascular system. The purpose of this study was to assess a possible association between HRV and ICG-based parameters of cardiac function in patients newly diagnosed with acromegaly. Methods: This observational cohort study was conducted on 33 patients (18 men, mean age of 47 years) newly diagnosed with acromegaly and no significant comorbidities. A correlation analysis (Spearman’s rank coefficient R) of the parameters assessed via ICG and the HRV assessed via 24 h ambulatory electrocardiography was performed. ICG assessments included the following parameters: stroke volume index (SI), cardiac index (CI), acceleration index (ACI), velocity index (VI), and Heather index (HI). The analysis of HRV included both time-domain parameters (pNN50, SDNN, SDSD, rMSSD) and frequency-domain parameters (total power (TP) and its individual frequency bands: low-frequency (LF day/night), high-frequency (HF day/night), and the LF/HF ratio (day/night)). Results: Frequency-domain HRV analysis showed the following correlations: (1) lower nighttime LF values with higher ACI (R = −0.38; *p* = 0.027) and HI (R = −0.46; *p* = 0.007) values; (2) higher nighttime HF values with higher ACI (R = 0.39; *p* = 0.027) and HI (R = 0.43; *p* = 0.014) values; (3) lower nighttime LF/HF values with higher ACI (R = −0.36; *p* = 0.037) and HI (R = −0.42; *p* = 0.014) values; (4) higher nighttime TP values with higher SI values (R = 0.35; *p* = 0.049). Time-domain parameters of HRV showed a significant correlation only between the nighttime values of SDSD and SI (R = 0.35; *p* = 0.049) and between the daytime and nighttime values of SDNN and HR (R = −0.50; *p* = 0.003 and R = −0.35; *p* = 0.046). In multivariate regression, only ACI was revealed to be independently related to HRV. Conclusions: In patients newly diagnosed with acromegaly, the relationship between the sympathetic–parasympathetic balance assessed via HRV and the hemodynamic profile assessed via ICG was revealed. Better function of the left ventricle was associated with a parasympathetic shift in the autonomic balance.

## 1. Introduction

Acromegaly (AC) is a rare chronic endocrine condition involving structural and functional cardiovascular abnormalities due to abnormal growth hormone (GH) and insulin-like growth factor 1 (IGF−1) levels [1]. Some of the most common causes of increased AC morbidity and mortality are cardiovascular complications, such as arrhythmias and sudden cardiac death [2,3,4]. Patients with AC are at a higher risk of developing arrhythmias than healthy individuals [5]. One anatomical predictor of arrhythmias in AC may be areas of myocardial fibrosis characteristic of acromegalic cardiomyopathy [6]. Therefore, each patient newly diagnosed with AC should undergo a comprehensive clinical evaluation, including a quantitative cardiovascular risk assessment [7]. Moreover, novel noninvasive predictors, which would reflect subclinical cardiovascular dysfunction in this group of patients, should be sought. A thorough clinical evaluation of patients with AC may be complemented by assessing balance in the autonomic nervous system (ANS), which plays an important role in regulating cardiovascular function [8,9,10,11]. There is a documented association between ANS imbalance and increased cardiovascular morbidity and mortality, including sudden cardiac death in patients with heart failure and coronary artery disease [12,13,14,15,16]. However, few studies have focused specifically on patients with AC [8,9,10,11]. Acromegaly may be expected to affect the interactions between the cardiovascular and autonomic nervous systems.

The autonomic nervous system dysfunction may cause adverse hemodynamic sequelae, which may lead to the development and progression of cardiovascular complications in patients with AC [17]. Finding a relationship between heart rate variability (HRV) and the hemodynamic profile in patients newly diagnosed with AC may help explain the association between ANS balance and both cardiovascular function and the risk of complications. A handful of studies in patients with AC indicate a better prognostic value of HRV analysis in comparison with conventional markers of the risk of cardiovascular death [8,9,10,11]. Hemodynamic disorders assessed via impedance cardiography (ICG) may be an important indicator of subclinical left ventricular dysfunction, which would help to stratify cardiovascular risk in the AC population [17]. The use of ICG and HRV in a comprehensive assessment of patients with AC helps evaluate the association between hemodynamic status and autonomic balance. This may prove to be a considerable added value in the diagnosis of subclinical cardiovascular dysfunction.

Therefore, the purpose of this study was to assess the possible association between HRV and impedance cardiographic markers of the heart’s function as a pump in patients with newly diagnosed AC and no significant comorbidities.

## 2. Materials and Methods

### 2.1. Study Population

This observational cohort study was conducted at the Military Institute of Medicine –National Research Institute. A total of 33 patients, hospitalized due to a new AC diagnosis, with no significant comorbidities (see exclusion criteria below), and no prior endocrine or surgical treatment, were enrolled prospectively. The study was conducted in accordance with the principles of the Declaration of Helsinki and Good Clinical Practice (GCP). All patients participating in this study signed an informed consent form. The study protocol was approved by the ethics committee of the Military Institute of Medicine–National Research Institute in Warsaw (approval No. 76/WIM/2016).

Acromegaly was diagnosed based on the European Society of Endocrinology (ESE) guidelines, which involve standard clinical, hormonal, and imaging criteria [18], such as the characteristic somatic symptoms and laboratory abnormalities: GH and IGF-1 levels above the normal levels for the sex and age and no GH suppression in a 75 g oral glucose tolerance test (OGTT) < 46 pmol/L (1.0 mcg/L). Magnetic resonance imaging (MRI) showed the presence of a neuroendocrine pituitary tumor in all patients. Hormonal assessment of pituitary function was expanded to include the levels of adrenocorticotropic hormone (ACTH), follicle-stimulating hormone (FSH), luteinizing hormone (LH), and thyroid-stimulating hormone (TSH). The patients were evaluated in terms of comorbid hypertension (HTN) and disorders of carbohydrate metabolism (type 2 diabetes mellitus (T2DM), impaired fasting glucose (IFG), and impaired glucose tolerance (IGT)).

The exclusion criteria were the following severe comorbidities that might affect cardivascular hemodynamics or HRV: heart failure with mildly reduced or reduced ejection fraction (<50%); chronic coronary syndrome; history of myocardial infarction, non-sinus rhythm (e.g., atrial fibrillation); permanent cardiac pacing; significant arrhythmias; a high number of artifacts or extrasystolic beats (>500/day) in an ambulatory electrocardiography (Holter); history of pulmonary embolism; chronic obstructive pulmonary disease; chronic kidney disease with an estimated glomerular filtration rate (eGFR) of <60 mL/min/1.73 m^2^ (calculated with the MDRD formula: male = 170 × (serum creatnine) − 0.999 × (age) − 0.176 × (serum urea) − 0.170 × (serum albumin) + 0.318; black male: MDRD × 1.180; female: MDRD × 0.76; black female: MDRD × 0.762 × 1.180) [19]; neurological conditions, including documented history of a stroke or transient ischemic attack (TIA); central nervous system pathologies, including multiple sclerosis, polyneuropathy, peripheral vascular disease, and respiratory failure; history of hormonal or neurosurgical treatment of a neuroendocrine pituitary tumor; post head trauma status; current pregnancy; and the lack of informed consent.

### 2.2. Clinical Evaluation

Each patient underwent a thorough clinical evaluation, including cardiovascular risk factors, cardiovascular symptoms, comorbidities, smoking, and family history of cardiovascular disease. The following parameters were measured: heart rate (HR), body mass index (BMI), the rate of HNT, and disorders of carbohydrate metabolism. Each patient underwent standard systolic blood pressure (SBP) and diastolic blood pressure (DBP) measurements with an automated blood pressure (BP) monitor (Omron M4 Plus, Kyoto, Japan) based on European Society of Cardiology (ESC) guidelines [20].

### 2.3. Impedance Cardiography

Impedance cardiography is a modern, noninvasive, and well-verified method for hemodynamic status assessment, allowing for the assessment of cardiovascular hemodynamic function, including vascular stiffness, the volume of blood in the vascular bed, and the function of the heart as a pump [21,22,23]. Each patient with AC underwent measurement of hemodynamic parameters via ICG with a Niccomo™ device (Medis, Ilmenau, Germany). The recorded ICG parameters and the SBP and DBP values, measured with an arm cuff by a trained nurse in 2 min intervals during a 10 min resting assessment in a recumbent position, were used to analyze hemodynamic indices of cardiovascular function. Thoracic impedance variations helped determine certain parameters of the heart’s function as a pump associated with the volumetric flow rate during left ventricular systole, such as stroke volume (SV (mL)), SV index (SI (mL/m^2^)), cardiac output (CO (mL/min)), and cardiac index (CI (mL × m^−2^ × min^−1^)). The impedance curve and the electrocardiogram (EKG) served as the basis for determining myocardial contractility parameters, such as the velocity index (VI (1 × 1000^−1^ × s^−1^)), reflecting the peak aortic blood flow, acceleration index (ACI (1/100/s^2^)), reflecting the peak acceleration of blood in the aorta, and Heather index (HI (Ohm/s^2^)), characterizing the inotropic function of the heart, and being the ratio of peak ventricular ejection flow to the time from the peak of the Q-point in the EKG to the Z-point in the ICG curve. We calculated the hemodynamic parameters associated with artery compliance based on concurrent SBP and DBP measurements: pulse pressure (PP), systemic vascular resistance (SVR (dyn × s × cm^−5^), SVR index (SVRI (dyn × s × cm^−5^ × m^2^)), total artery compliance (TAC (mL/mm Hg), and the TAC index (TACI (mL/mm Hg × m^2^)). Moreover, we assessed the parameter reflecting the total fluid content (TFC (1/kOhm)). High-cardiovascular-risk groups were identified based on the PREDICT study, adopting the cut-off values for SI at <35 mL/m^2^ and for TFC at >35 1/kOhm [24].

### 2.4. Heart Rate Variability Assessment

Heart rate variability assessment is a noninvasive, well-documented method for monitoring the effect of the ANS sympathetic and parasympathetic function on heart rate regulation [25]. Each patient with AC underwent 24 h electrocardiographic monitoring (Holter) with 3-channel digital LifeCard CF recorders (Spacelabs Healthcare, St Snoqualmie, WA, USA). Prior to this assessment, all patients were advised to avoid physical exertion, smoking, and alcohol, and to rest between 10 pm and 6 am. The assessments were conducted in a hospital setting to limit the possible effect of other confounding factors. The minimum, mean, and maximum HR, any arrhythmias and conduction blocks, and HRV were assessed. The Pathfinder SL system, HRV Advanced Option (Spacelabs Healthcare; St Snoqualmie, WA, USA), was used for the analysis of the time-domain and frequency-domain parameters of HRV. During an initial assessment of EKG, any incorrectly classified beats were re-classified, artifacts were eliminated, and the recordings were analyzed for any arrhythmias, conduction blocks, or ST segment changes.

We analyzed R–R intervals between normal QRS complexes, excluding the intervals before and after extrasystolic beats. EKGs with a large number of artifacts and extrasystolic beats (>500/day) were excluded from the analysis.

Automatic time-domain analysis included HRV parameters from the day, the night, and the whole 24 h period. The time-domain analysis of HRV included the proportion of over 50 ms differences between adjacent R–R (NN) intervals (pNN50) [%], the standard deviation from the mean NN interval (SDNN) [ms], the standard deviation of successive NN interval differences (SDSDs), and the square root of the mean sum of the squared differences between adjacent NN intervals (rMSSD) [ms]. The SDNN parameter was found to reflect long-term HRV, particularly reflecting parasympathetic ANS function. The rMSSD and pNN50 parameters were found to show parasympathetic ANS function [25,26,27,28].

The frequency-domain analysis of HRV was conducted with the use of the Fast Fourier Transform (FFT). The frequency-domain analysis of HRV included the variance of all NN intervals (total power, TP) and its individual frequency bands: low-frequency oscillations of 0.05–0.15 Hz (LF day/night), high-frequency of 0.15–0.35 Hz (HF day/night), and the LF/HF day/night ratio. A complete analysis included all these variables during a 24 h period, during the day and at night. Heart rate variability was presented graphically in the form of a histogram. The HF parameter was found to be an indicator of parasympathetic function, and the LF parameter was found to depend on both the sympathetic and parasympathetic function. The relationship between LF and HF (the LF/HF ratio) was found to reflect the sympathetic–parasympathetic balance [25,26,27,28].

### 2.5. Statistical Methods

The statistical analysis of the obtained data was conducted with Statistica 12.0 software (StatSoft Inc., Tulsa, OK, USA). Continuous variables were expressed as means ± standard deviation (SD), medians, and interquartile intervals; and categorical (qualitative) variables were expressed as absolute values (n) and proportions (%). The distribution of continuous variables was assessed visually and with the use of the Shapiro–Wilk test.

The distribution and normality of data were assessed visually and with the use of the Kolmogorov–Smirnov test. Continuous variables have been expressed as means and SDs, whereas nominal categorical variables have been expressed as absolute numbers and proportions. Possible correlations between the variables were evaluated with Pearson and Spearman correlation. Additionally, multivariable regression models, including potential confounders (age, BMI, and HR), to verify the magnitude of associations for identified univariate correlations between impedance and HRV parameters were analyzed. *p*-values of <0.05 were considered statistically significant.

## 3. Results

### 3.1. Baseline Characteristics

Study group characteristics are presented in Table 1. The study group comprised young and middle-aged individuals (47.0 ± 13.5 years of age) newly diagnosed with active AC who received no prior hormonal or neurosurgical treatment. Eighteen patients (54.5%) were previously diagnosed with HTN. All of them received antihypertensive treatment in the form of monotherapy or combined two-drug therapy and had well-controlled HTN. The mean BP value was 121/77 mm Hg. All patients had a preserved left ventricular ejection fraction of approximately 64.7%. A total of 6 out of 33 patients with AC (18.2%) had comorbid T2DM. Two of those patients were treated with metformin alone, three received metformin and insulin, and one was treated with diet. Detailed characteristics of this study population have been already published [18].

### 3.2. Relationship between the HRV Metrics and the ICG Parameters

The time-domain analysis of HRV showed a significant correlation only between the night values of SDSD and SI (R = 0.35; *p* = 0.049) and between the daytime and nighttime values of SDNN and HR (R = −0.50; *p* = 0.003 and R = −0.35; *p* = 0.046). Detailed results are presented in Table 2. No additional significant correlations have been observed between any other time-domain measurements of HRV (SDNN, rMSSD, pNN50) and the hemodynamic profile parameters assessed via ICG (SI, VI, ACI, HI).

**Figure 1:**
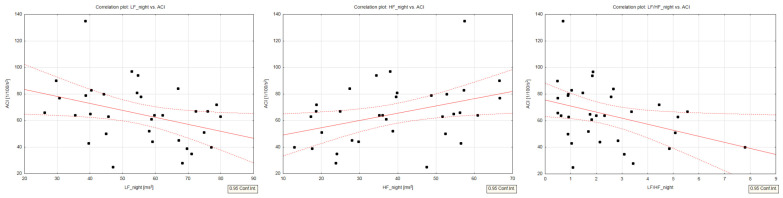
Correlation plots for ACI vs. LF_night (**left chart**, R = −0.38; *p* = 0.027), HF_night (**middle chart**, R = 0.39; *p* = 0.027), and LF/HF (**right chart**, R = −0.36; *p* = 0.037). (Point and line graph—graphical representation of strength and direction of correlation).

The relationship between the increased sympathetic tone and worse left ventricular performance was noted. Nighttime frequency-domain HRV analysis revealed the following correlations: (1) lower LF values with higher ACI (R = −0.38; *p* = 0.027) and HI (R = −0.46; *p* = 0.007) values; (2) higher HF values with higher ACI (R = 0.39; *p* = 0.027) and HI (R = 0.43; *p* = 0.014) values; (3) lower LF/HF values with higher ACI (R = −0.36; *p* = 0.037) and HI (R = −0.42; *p* = 0.014) values; and (4) higher TP values with higher SI values (R = 0.35; *p* = 0.049). Detailed results are presented in Table 3 and Figure 1. In multivariate regression models, significant partial correlations, independent from age, BMI, and HR, were noted for ACI with nighttime LF (R = −0.47; *p* = 0.037), nighttime HF (R = −0.46; *p* = 0.040), and nighttime LF/HF (R = −0.38; *p* = 0.039), while HI and SI were revealed not to be independently correlated. Men and women presented no differences in frequency-domain or time-domain HRV parameters.

## 4. Discussion

The results of our study support the existence of a relationship between HRV parameters and the hemodynamic profile in patients newly diagnosed with active AC with no clinically significant comorbidities. Better left ventricular function as a pump was associated with a shift in autonomic balance toward parasympathetic activity. Already at the subclinical stage, patients with newly diagnosed AC may have cardiovascular hemodynamic abnormalities associated with an imbalance of the ANS with a shift in balance towards sympathetic activity.

Patients with AC constitute a group with high cardiovascular risk, including the development of arrhythmias [2,3,4,5]. Parolin et al. [29] pointed out that AC patients are burdened with increased rates of premature ventricular beats and impaired preclinical markers of arrhythmia, including reduced HRV, increased late potentials, and QT interval dispersion. These markers are associated with an adverse cardiovascular prognosis in the general population [30]. There have been reported cases of sudden cardiac death, ventricular arrhythmia, and advanced atrioventricular block in patients with AC [29]. Orosz et al. [31] demonstrated a significantly increased short-term QT interval inter-beat variability in patients with AC, despite unchanged conventional parameters of ventricular repolarization. This increased time-domain QT interval variability may be an early indicator of an increased risk of arrhythmia. The relationship between arrhythmias and autonomic imbalance (with a shift towards sympathetic activity) is a commonly accepted phenomenon [32,33,34].

The study population comprised young and middle-aged patients newly diagnosed with active AC, with no comorbidities that might significantly impair cardiovascular function. The patients included in the study had not received prior hormonal or neurosurgical treatment for a neuroendocrine pituitary tumor. All patients had normal left ventricular systolic function, assessed echocardiographically. Patients with AC who already had significant cardiovascular dysfunction or had multiple comorbidities that might affect the results obtained in this study were excluded. Consistent with previous reports, 20% of our study population had T2DM, and 25% had HTN [35,36,37,38,39]. Although both of these comorbidities may affect the cardiovascular system, several authors reported AC to be significantly associated with autonomic dysfunction of the heart, irrespective of T2DM and HTN [8,10,11]. Therefore, the methodology adopted for our study ensures a reliable, relatively unconfounded assessment of the effect of AC on the combined function of the autonomic and cardiovascular systems. It is worth noting that a thorough evaluation of cardiovascular hemodynamic parameters (via ICG) and ANS balance as markers of subclinical cardiovascular dysfunction is—to the best of our knowledge—the first attempt to use both methods simultaneously in a group of patients with AC.

The time-domain analysis of HRV showed a correlation between SDSD and SI (positive) and SDNN and HR (negative). This may indicate a relationship between increased pulse and increased sympathetic activity; however, the relationship with SI is likely a secondary phenomenon. We observed no correlation for any other, more commonly used, time-domain HRV indices (rMSSD, pNN50). We would like to emphasize that hyperkinetic circulation may be the first phase of subclinical acromegalic cardiomyopathy, which increases the risk of left ventricular dysfunction and the development of fully symptomatic heart failure; this is consistent with earlier reports [36,39,40].

Particularly interesting were our results on the correlation of frequency-domain HRV parameters during the night. We observed a significant relationship between increased parasympathetic activity and the values of parameters reflecting the function of the left ventricle as a pump, primarily the parameters specific to ICG, namely HI and ACI. These findings suggest that at an early stage of AC, before advanced complications develop, there is already cardiovascular hemodynamic dysfunction associated with an adverse ANS imbalance. Our findings are consistent with those reported by other authors. Relevant literature data indicate that ANS dysfunction (involving increased sympathetic activity and reduced parasympathetic activity) plays an important role in the pathophysiology of heart failure, coronary artery disease, and HTN and is an important factor in the development of cardiovascular complications [12,13,14,15,16]. Other studies that used HRV analysis in patients with cardiovascular conditions showed an increased sympathetic activity already in the early stages of these conditions, with a greater degree of ANS dysfunction observed in individuals with preexisting organ complications [16,27]. Autonomic imbalance of the heart is commonly believed to be an underlying cause of cardiovascular morbidity and mortality [32,41]. Long-term sympathetic hyper-reactivity leads to long-term hemodynamic consequences, resulting in cardiac dysfunction and an increased risk of sudden cardiac death and ventricular arrhythmias [32,33,34]. Some studies suggest a greater prognostic value of HRV analysis than that of conventional biomarkers of the risk of cardiovascular death [32,33,34,41].

Although recent years saw improvements in the treatment of AC and the associated comorbidities, cardiovascular complications remain one of the key causes of morbidity and premature mortality in AC [42]. The main predictors of increased mortality in patients with AC are initial GH and IGF-1 levels, age, disease duration, and cardiovascular conditions [7,42,43]. The most recent studies showed that excessive GH/IGF-1 has adverse effects on the cardiovascular system not only via increased expression of GH and IGF-I receptors in cardiomyocytes or enhanced sensitivity of myocardial myofilaments to calcium, but also by inducing endothelial dysfunction, which fosters a pro-atherogenic environment and a systemic inflammatory reaction [42,44,45,46,47,48].

Moreover, the adverse cardiovascular profile of patients with AC may be associated with higher rates of sleep apnea and sleep hypopnea [49], which is consistent with our observations of clearer correlations between the nighttime parameters in a time-domain HRV analysis. However, the pathophysiology of ANS dysfunction and hemodynamic disturbances in this patient population still remains unclear. One of our earlier studies showed the presence of subclinical cardiovascular hemodynamic dysfunction in the form of hyperkinetic circulation and reduced markers of cardiac contractility already at the time of AC diagnosis [17]. A few existing studies demonstrated sympathetic–parasympathetic imbalance in patients with AC [8,9,10,11]. Like in our study, one controlled trial on HRV, conducted both with time-domain and frequency-domain parameters in patients newly diagnosed with AC, showed autonomic imbalance with enhanced sympathetic tone [10]. That study demonstrated that such HRV parameters as SDNN (*p* = 0.001), SDANN (*p* = 0.001), rMSSD (*p* = 0.001), pNN50 (*p* = 0.001), and HF (*p* = 0.001) were significantly reduced in patients with AC, whereas LF (*p* = 0.046) and LF/HF (*p* = 0.001) were significantly higher in AC patients. The study also showed significant negative correlations between disease duration and pNN50, rMSSD, and SDNN. A multivariate linear regression analysis showed AC to be significantly associated with autonomic cardiac dysfunction, irrespective of T2DM. After patients with T2DM were excluded from analysis, the autonomic function of the heart was impaired in patients with AC in comparison with that in healthy controls [10].

Italian studies [8] showed the time-domain parameters SDNN and SDANN to be considerably reduced both in patients with AC and T2DM and in patients with AC without T2DM in comparison with those in controls. An inverse correlation was found between plasma glucose levels and each of the following: pNN50, SDNN, SDNN index, and the standard deviation of 5 min average NN intervals (SDANN). Compared with the control group, patients with AC showed higher rates of arrhythmias and higher rates of late potentials, with a significant correlation with Lown’s grading system of ventricular premature beats. Patients with significant ventricular arrhythmia showed decreased time-domain HRV parameters in comparison with patients without significant arrhythmia. The authors of that study emphasized that patients with AC at a higher risk for arrhythmia also suffer from autonomic imbalance [8]. Resmini et al. [11] evaluated frequency-domain HRV parameters in patients with AC and showed significantly lower LF/HF ratios, measured both in a recumbent position (*p* = 0.002) and in a standing position (*p* < 0.001), in comparison with those in healthy individuals. HF while standing was significantly higher in patients with AC than in healthy individuals (*p* < 0.001) and patients with type 1 diabetes mellitus (*p* = 0.004), but did not differ from that in patients with T2DM (*p* = 0.069). Therefore, the available data indicate that autonomic imbalance in patients with AC may be an early marker of abnormal interaction between the cardiovascular system and the ANS, which seems to be confirmed by subclinical cardiovascular hemodynamic dysfunction in patients newly diagnosed with AC [8,9,10,11,17].

### 4.1. Clinical Implications

Sympathetic abnormalities in patients with AC may be a novel cardiovascular risk factor [11]. The use of modern, noninvasive diagnostic methods increases the chance of detecting subclinical and mildly symptomatic abnormalities, which facilitates personalized therapy focused on cardiovascular risk reduction. The observed association between HRV and the hemodynamic profile suggests new treatment goals, including methods modulating ANS function. It is worth noting that the modern noninvasive methods of cardiovascular assessment used in this study may be easily used both in hospital and outpatient settings to assess patients with AC.

### 4.2. Limitations

The main limitation of our study was the relatively small sample size. This was a result of both the low incidence of AC caused by neuroendocrine tumors of the pituitary and the adopted exclusion criteria. Nonetheless, the study group became more homogenous as a result, and the potentially confounding effect of any comorbidities on the ANS and circulatory hemodynamics was greatly limited. While interpreting the results, the potential effect of antihypertensive therapy should be considered. Further multi-center prospective studies are needed to elucidate the precise pathophysiological mechanisms behind ANS imbalance and cardiovascular hemodynamic dysfunction in patients with AC and the effect of those factors on the development of cardiovascular complications.

## 5. Conclusions

This study showed a relationship between the sympathetic–parasympathetic balance assessed via HRV and the hemodynamic profile assessed via ICG in patients newly diagnosed with AC. The better left ventricular function as a pump was associated with a shift in autonomic balance towards increased parasympathetic activity. The results of this study encourage further studies to evaluate their clinical and prognostic significance. Already at the subclinical stage, patients with newly diagnosed AC may have cardiovascular hemodynamic abnormalities associated with an imbalance of the ANS with a shift in balance towards sympathetic activity.

## Figures and Tables

**Table 1:** Baseline characteristics of the study group with acromegaly.

VARIABLE	Mean ± SD (Median; Interquartile Interval) or n (%)
DEMOGRAPHIC DATA	
Age, years	47.0 ± 13.5 (47.0; 38.0–61.0)
Male sex	18 (54.5)
BMI, kg/m^2^	27.8 ± 4.1 (27.7; 25.3–30.1)
BMI 18.5–24.9, kg/m^2^	7 (21.2)
BMI 25–29.9, kg/m^2^	17 (51.5)
BMI ≥ 30, kg/m^2^	9 (27.3)
HR, bpm	75.6 ± 10.7 (77.0; 67.0–82.0)
SBP, mm Hg	121.0 ± 11.2 (123.0; 115.0–127.0)
DBP, mm Hg	77.0 ± 9.7 (77.0; 72.0–81.0)
CLINICAL DATA	
HTN	18 (54.5)
T2DM	6 (18.2)
Prediabetes	10 (33.3)
Creatinine, mg/dL	0.76 ± 0.20 (0.80; 0.60–0.80)
LVEF, %	62.5 ± 5.1 (62.9; 60.8–66.0)

Abbreviations: BMI—body mass index; DBP—diastolic blood pressure; HTN—hypertension; HR—heart rate; LVEF—left ventricular ejection fraction; SBP—systolic blood pressure; T2DM—type 2 diabetes mellitus.

**Table 2:** Correlations between the time-domain parameters of heart rate variability and the parameters measured via impedance cardiography in patients with acromegaly.

	Correlations: R (p)							
VARIABLE	SDNN Day	rMSSD_day	SDSD_day	pNN50_day	SDNN_night	rMSSD_night	SDSD_night	pNN50_night
Age	0.14 (0.445)	−0.39 (0.028)	−0.34 (0.059)	−0.42 (0.016)	−0.22 (0.228)	−0.34 (0.056)	−0.37 (0.038)	−0.28 (0.109)
BMI	0.27 (0.138)	0.124 (0.499)	0.121 (0.511)	0.27 (0.131)	0.19 (0.290)	−0.11 (0.559)	0.19 (0.302)	0.16 (0.359)
HR, bpm	−0.50 (0.003)	−0.16 (0.381)	−0.14 (0.428)	−0.20 (0.264)	−0.35 (0.046)	−0.19 (0.274)	−0.21 (0.251)	−0.21 (0.246)
SBP, mm Hg	0.13 (0.490)	0.26 (0.158)	0.28 (0.123)	0.13 (0.457)	0.06 (0.739)	0.08 (0.666)	0.08 (0.648)	0.09 (0.060)
DBP, mm Hg	0.004 (0.982)	0.01 (0.945)	0.02 (0.918)	−0.09 (0.585)	−0.07 (0.698)	−0.01 (0.934)	−0.03 (0.869)	0.01 (0.952)
MBP, mm Hg	−0.007 (0.967)	0.001 (0.994)	0.01 (0.933)	−0.11 (0.531)	−0.11 (0.555)	−0.03 (0.851)	−0.05 (0.777)	0.0008 (0.996)
PP, mm Hg	0.13 (0.472)	0.19 (0.301)	0.23 (0.195)	0.18 (0.313)	−0.10 (0.569)	−0.07 (0.699)	−0.03 (0.858)	−0.07 (0.708)
SI, mL/m^2^	0.16 (0.385)	0.14 (0.427)	0.14 (0.439)	0.26 (0.147)	0.28 (0.124)	0.33 (0.064)	0.35 (0.049)	0.27 (0.129)
CI, mL × m^−2^ × min^−1^	−0.16 (0.387)	0.02 (0.909)	0.03 (0.860)	0.11 (0.553)	0.03 (0.888)	0.19 (0.287)	0.21 (0.252)	0.12 (0.493)
VI, 1 × 1000^−1^ × s^−1^	−0.03 (0.854)	−0,05 (0.788)	−0.03 (0.868)	0.01 (0.944)	0.13 (0.467)	0.17 (0.354)	0.16 (0.384)	0.11 (0.529)
ACI, 1/100/s^2^	−0.07 (0.709)	0.19 (0.289)	0.20 (0.266)	0.16 (0.363)	−0.03 (0.879)	0.24 (0.186)	0.19 (0.291)	0.16 (0.366)
HI, Ohm/s^2^	−0.05 (0.792)	0.03 (0.871)	0.07 (0.682)	−0.04 (0.822)	−0.07 (0.721)	0.09 (0.605)	0.08 (0.657)	0.05 (0.773)
SVRI, dyn × s × cm^−5^ × m^2^	0.12 (0.497)	−0.01 (0.966)	−0.02 (0.929)	−0.13 (0.473)	−0.09 (0.591)	−0.16 (0.391)	−0.18 (0.333)	−0.13 (0.473)
TACI, mL/mmHg × m^2^	0.0001 (0.999)	−0.07 (0.719)	−0.10 (0.587)	0.08 (0.668)	0.20 (0.289)	0.34 (0.074)	0.34 (0.073)	0.27 (0.153)
TFC, 1/kOhm	0.005 (0.977)	0.17 (0.351)	0.11 (0.539)	0.31 (0.082)	0.23 (0.212)	0.17 (0.347)	0.15 (0.425)	0.19 (0.267)

Abbreviations: ACI—acceleration index; CI—cardiac index; DBP—diastolic blood pressure; HI—Heather index; HR—heart rate; MBP—mean blood pressure; PP—pulse pressure; SBP—systolic blood pressure; SI—stroke index; SVRI—systemic vascular resistance index; TAC—total artery compliance; TFC—thoracic fluid content; VI—velocity index; pNN50—proportion of NN50 divided by total number of NNs; SDNN—standard deviation of the NN interval; SDSD—standard deviation of successive differences; rMSSD—square root of the mean of the sum of the squares of differences between adjacent NN intervals.

**Table 3:** Correlations between the frequency-domain parameters of heart rate variability and the parameters measured via impedance cardiography in patients with acromegaly.

	Correlations: R (p)							
VARIABLE	LF_day	LF_night	HF_day	HF_night	LF/HF_day	LF/HF_night	TP_day	TP_night
Age	−0.37 (0.032)	−0.05 (0.778)	0.34 (0.056)	−0.02 (0.930)	−0.40 (0.023)	−0.05 (0.789)	−0.39 (0.026)	−0.41 (0.020)
BMI	−0.01 (0.935)	0.16 (0.386)	0.01 (0.982)	−0.11 (0557)	0.05 (0.768)	0.16 (0.384)	0.26 (0.143)	0.24 (0.195)
HR, bpm	0.06 (0.747)	−0.12 (0.517)	−0.217 (0.224)	0.001 (0.995)	0.14 (0.419)	0.001 (0.994)	−0.38 (0.033)	−0.25 (0.163)
SBP, mm Hg	−0.18 (0.311)	−0.06 (0.721)	0.187 (0.295)	0.02 (0.922)	−0.13 (0.481)	−0.120 (0.505)	0.11 (0.536)	0.15 (0.417)
DBP, mm Hg	−0.09 (0.604)	−0.04 (0.835)	0.01 (0.953)	−0.03 (0.878)	−0.02 (0.901)	−0.01 (0.949)	−0.07 (0.718)	0.01 (0.961)
MBP, mm Hg	−0.14 (0.444)	−0.09 (0.587)	0.06 (0.727)	0.02 (0.918)	−0.07 (0.705)	−0.08 (0.672)	−0.08 (0.669)	−0.004 (0.981)
PP, mm Hg	−0.26 (0.135)	−0.09 (0.612)	0.33 (0.06)	0.04 (0.838)	−0.252 (0.157)	−0.13 (0.464)	0.17 (0.359)	−0.03 (0.868)
SI, mL/m^2^	0.08 (0.672)	−0.181 (0.313)	0.10 (0.557)	0.27 (0.128)	−0.03 (0.864)	−0.24 (0.175)	0.25 (0.165)	0.35 (0.049)
CI, mL × m^−2^ × min^−1^	0.11 (0.554)	−0.32 (0.069)	−0.02 (0.908)	0.32 (0.071)	0.05 (0.781)	−0.28 (0.108)	0.000 (1.000)	0.18 (0.331)
VI, 1 × 1000^−1^ × s^−1^	−0.01 (0.962)	−0.27 (0.121)	0.16 (0.357)	0.322 (0.067)	−0.16 (0.383)	−0.29 (0.103)	−0.11 (0.537)	0.10 (0.598)
ACI, 1/100/s^2^	−0.08 (0.636)	−0.38 (0.027)	0.19 (0.291)	0.38 (0.026)	−0.19 (0.279)	−0.36 (0.037)	0.16 (0.379)	0.09 (0.610)
HI, Ohm/s^2^	−0.22 (0.214)	−0.46 (0.007)	0.28 (0.109)	0.42 (0.014)	−0.33 (0.059)	−0.42 (0.014)	0.007 (0.967)	−0.06 (0.749)
SVRI, dyn × s × cm^−5^ × m^2^	−0.16 (0.359)	0.18 (0.318)	0.06 (0.716)	−0.21 (0.233)	−0.09 (0.632)	0.17 (0.349)	−0.03 (0.851)	−0.16 (0.380)
TACI, mL/mmHg × m^2^	0.22 (0.238)	−0.26 (0.169)	−0.06 (0.738)	0.34 (0.067)	0.11 (0.561)	−0.25 (0.181)	0.04 (0.834)	0.31 (0.09)
TFC, 1/kOhm	0.21 (0.231)	0.23 (0.194)	−0.27 (0.130)	−0.17 (0.331)	0.33 (0.06)	0.19 (0.277)	0.04 (0.841)	0.28 (0.124)

Abbreviations: ACI—acceleration index; CI—cardiac index; DBP—diastolic blood pressure; HI—Heather index; HR—heart rate; MBP—mean blood pressure; PP—pulse pressure; SBP—systolic blood pressure; SI—stroke index; SVRI—systemic vascular resistance index; TAC—total artery compliance; TFC—thoracic fluid content; VI—velocity index; TP–total power; LF—low frequency; HF—high frequency; LF/HF—the ratio of low-frequency oscillations to high-frequency oscillations.

## Data Availability

The data presented in this study are available upon request from the corresponding author. The data are not publicly available due to privacy or ethical restrictions.
